# Gut feelings, deliberative thought, and paranoid ideation: A study of experiential and rational reasoning

**DOI:** 10.1016/j.psychres.2011.12.031

**Published:** 2012-05-15

**Authors:** Daniel Freeman, Nicole Evans, Rachel Lister

**Affiliations:** Department of Psychiatry, Oxford University, UK

**Keywords:** Delusions, Paranoia, Reasoning, Experiential, Rational, Personality

## Abstract

Rapid intuitive hunches or gut feelings may be a compelling source of evidence for paranoid ideas. Conversely, a failure to apply effortful analytic thinking may contribute to the persistence of such thoughts. Our main aim was to examine for the first time the associations of persecutory thinking with experiential and rational thinking styles. Five hundred individuals recruited from the general population completed self-report assessments of current persecutory ideation, general reasoning styles and personality traits. Persecutory ideation was independently associated with greater use of experiential reasoning and less use of rational reasoning. The correlations were small. Persecutory ideation was also positively associated with neuroticism and negatively correlated with extraversion, agreeableness and conscientiousness. There was no evidence of an interaction between neuroticism and experiential reasoning in the prediction of paranoia, but high experiential reasoning in the context of low rational reasoning was particularly associated with persecutory ideation. Overall, the study provides rare evidence of self-reported general reasoning styles being associated with delusional ideation. Perceived reliance on intuition is associated with paranoid thinking, while perceived reliance on deliberation is associated with fewer such thoughts. The dual process theory of reasoning may provide a framework to contribute to the understanding of paranoid thinking.

## 1 Introduction

The process of making and revising judgements is of obvious importance in understanding delusional beliefs. Two parallel systems are considered to underlie decision-making: an effortless, bounded rationality, rapid judgement and a slow, reflective, conscious, analytic approach (e.g. Epstein, 1994; Evans and Over, 1996; Sloman, 1996; Stanovich, 1999). The types of reasoning within dual process theory have been called experiential and rational (or intuitive and reflective). Experiential reasoning, in contrast to rational reasoning, is viewed as being closely tied to affect, and hence particularly compelling. Emotional feelings are considered a key source of information for rapid judgements that can outweigh more considered evaluations, which is also seen in the related theoretical concepts of ‘risk-as-feeling’ and ‘the affect heuristic’ (Loewenstein et al., 2001; Slovic et al., 2002). It is our contention based upon clinical experience that quick decision-making based upon feelings of fear are a significant proximal contributor to the occurrence of paranoid thoughts. Individuals are closely following their anxious physiological reactions, or gut feelings, when suspicious thoughts come to mind. This clinical impression is consistent with theoretical and empirical work indicating that individuals with delusions often need to make decisions about confusing and ambiguous experiences, that they have a tendency to jump to conclusions, and anxiety is a predictor of paranoid thought occurrence (e.g. Garety and Freeman, 1999; Kapur, 2003; Freeman, 2007; Bentall et al., 2009; Ben-Zeev et al., 2010).

A self-report questionnaire of experiential (e.g. ‘I believe in trusting my hunches’, ‘I like to rely on my intuitive impressions’) and rational (e.g. ‘I have no problem thinking things through carefully’ ‘Using logic usually works well for me in figuring out problems in my life’) reasoning has been developed (Epstein et al., 1996; Pacini and Epstein, 1999). The Rational Experiential Inventory (REI) has rarely been examined in relation to psychiatric problems, and has typically been employed in studies of students. Of relevance to the current investigation, experiential thinking has been modestly positively correlated with the occurrence of paranormal and superstitious beliefs and schizotypal traits, with rational thinking showing an opposite pattern (e.g. Wolfradt et al., 1999; Aarnio and Lindeman, 2005; Genovese, 2005; Marks et al., 2008). The thinking styles have also been studied in relation to the ‘Big Five’ personality traits (McCrae and Costa, 1987). The results indicate similar small positive correlations of the two thinking styles to the different personality traits, except for neuroticism for which experiential reasoning has not been found to be associated while rational thinking is typically inversely correlated (e.g. Pacini and Epstein, 1999; Marks et al., 2008; Witteman et al., 2009). It is of note for establishing the independence of rational and experiential styles that they typically do not correlate with each other (e.g. Pacini and Epstein, 1999; Wolfradt et al., 1999; Aarnio and Lindeman, 2005), meaning that different combinations of the two styles can be used by individuals.

Our aim in this study was to examine experiential and rational thinking in relation to persecutory thoughts specifically. As this was the first examination of the issue we used the method of a cross-sectional investigation in a large non-clinical adult population, based upon the accumulating evidence that each of the separate psychotic experiences is represented by continuous traits in the general population (e.g. van Os et al., 2009; Freeman et al., 2010b). It was predicted that paranoid thoughts would be positively associated with experiential reasoning and negatively associated with rational reasoning. The combination of experiential thinking without rational thinking was predicted to be associated with the most paranoid thinking. We also took the opportunity to examine for the first time the relationship of personality to persecutory thinking. We predicted that neuroticism and persecutory thinking would be positively correlated, because of the many studies indicating associations of anxiety and depression with paranoia (e.g. Freeman et al., 2008; Thewissen et al., 2011). Further we predicted that a reliance on experiential thinking in the context of neuroticism (i.e. negative fearful feelings) would particularly be associated with paranoid thinking. A clinical importance of pursuing this line of inquiry is that it has the potential to provide a framework, and a language, to use with patients in understanding how they came to paranoid interpretations of events.

## 2 Method

### 2.1 Participants

500 people from the city of Oxford took part in the study. The data were collected during the screening phase for an on-going experimental study (for which only approximately 10% of respondents will be eligible). Our team sent leaflets to local postcodes with the wording:

“*Volunteers Required for Psychological Research. We are looking for volunteers to take part in a medical research study being carried out at the university. The research would take three hours and you would be compensated for your time. If you would like to hear more about the research, then please contact us. We send detailed information about the study so that you can consider whether you would like to take part.”*

The individuals who responded were then invited to take part in the screening stage. Depending on participant preference, the screening questionnaires were either sent in the post or were made available via a web-link. According to the English Index of Multiple Deprivation 2010, Oxford ranks 131st out of 354, placing it in the top half of most deprived local authority areas in England. The unemployment rate in Oxford is approximately 6.3%, below the national average of around 7.7%. Half of the city's jobs are in the public sector and universities (double the average national rate).

### 2.2 Measures

#### 2.2.1 Paranoid Thoughts Scale Part B (GPTS-B; Green et al., 2008)

The GPTS-Part B measures persecutory ideation, as defined by Freeman and Garety (2000), over the past month. Each of the sixteen items in the scale (e.g. ‘Certain individuals have had it in for me’ ‘People have been hostile towards me on purpose’ ‘I was sure someone wanted to harm me’ ‘I was convinced there was a conspiracy against me’) are rated by the person on a 5-point scale (1–5). Scores can range from 16 to 80, with 16 indicating the absence of persecutory ideation and higher scores indicating greater persecutory ideation. The questionnaire has shown good psychometric properties in both clinical and non-clinical populations, and been validated against an experimental assessment of the occurrence of paranoid thinking (Freeman et al., 2008; Freeman et al., 2010b). In the present study the Cronbach's alpha of the scale was .95.

#### 2.2.2 Rational-experiential inventory (REI; Pacini and Epstein, 1999)

The REI is a 40-item measure of an individual's preference for two different thinking styles: rational and experiential. Each style is assessed using 20-item scales. Each can be further broken down into 10-item subscales, assessing self-evaluated ability in the given style (ability subscales) and reliance on and enjoyment of the given style (engagement subscales). Items are rated on a five point Likert scale where 1 is “completely false” and 5 is “completely true”. Examples of items from the rational scale include, “I have a logical mind” (rational ability) and “I enjoy solving problems that require hard thinking” (rational engagement). Examples from the experiential scale include, “I trust my initial feelings about people” (experiential ability) and “I often go on my instincts when deciding on a course of action” (experiential engagement). Mean scores for each subscale can range from 1 to 5, with 1 indicating a low ability/engagement and 5 indicating a high ability/engagement for each thinking style. Internal consistencies for the both the rational and experiential scales, and all four ability and engagement subscales are high (e.g. Björklund and Bäckström, 2008), as is the test-retest reliability of the scale (e.g. Handley et al., 2000). In the present study the Cronbach's alpha for the experiential scale was .78, and for the rational scale it was .73.

#### 2.2.3 Newcastle personality assessor (NPA; Nettle, 2007)

The NPA is a brief measure of personality. Five dimensions of personality (extraversion, neuroticism, conscientiousness, agreeableness and openness) are assessed by a total of 12 items rated on 5-point scales, with 1 being “very uncharacteristic”, 3 being “moderately characteristic” and 5 being “very characteristic”. Example items include: “planning parties and social events” (extraversion), “feeling stressed or worried” (neuroticism), “preparing for things well in advance” (conscientiousness), “making sure others are comfortable and happy” (agreeableness), and “thinking about philosophical or spiritual questions” (openness). Scores for each personality dimension are formed by summing the scores from the relevant two or three items. Higher scores indicate a higher level of the personality trait. The NPA dimension scores correlate with coefficients of .7 or higher with those in the International Personality Item Pool (Goldberg et al., 2006).

### 2.3 Analysis

Analyses were carried out using SPSS Version 19.0 (IBM, 2010). There were hardly any missing data (0.4%), because incomplete questionnaire responses were prevented for those using the website. 86% of participants did not have any missing data, while only an average of 3.2% was missing from the other 14% of participants. Where one or two items in a scale were missing these scores were prorated. The Paranoid Thoughts Scale scores were transformed (log to the base 10) to reduce significant skew, and the transformed data were used in the analyses. No other variable needed to be transformed. Summary mean scores of the measures were calculated and basic associations with demographic variables examined using Pearson correlations and analysis of variance. Associations between paranoia, reasoning style and personality were then initially examined using Pearson correlation coefficients. A multiple regression analysis was then used to test the ability of experiential and rational thinking to predict paranoia scores, which was repeated controlling for demographic variables. Analysis of variance was used to test for an interaction between experiential reasoning and neuroticism. In a secondary analysis to test for potential combinations of reasoning style we constructed four groups (see Epstein, 1998; Wolfradt et al., 1999): high experiential/low rational, high experiential/high rational, low experiential/low rational, low experiential/high rational. Participants were allocated into high or low rational or experiential thinking based on whether they were above or below the 50th percentile on each scale. The four groups were tested for differences in levels of paranoia and neuroticism using analysis of variance, with post hoc least significant difference tests. Significance test results for all the analyses are quoted as two-tailed probabilities.

## 3 Results

### 3.1 Demographic information

The participant group comprised 210 males and 290 females, with a mean age of 45.7 years (S.D. = 20.2). 199 participants were single, 167 were married or in a civil partnership, 50 were divorced, 47 were co-habiting, and 32 people were widowed. The ethnicities of the group were White (*n* = 447), Black Caribbean (*n* = 3), Black African (*n* = 6), Indian (*n* = 7), Pakistani (*n* = 6), Chinese (*n* = 4), and other (*n* = 25). The highest level of education reached by participants were none/GCSE (n = 50), A-level (*n* = 135), degree (*n* = 180) and postgraduate (*n* = 134). 81 participants reported having been diagnosed or treated for a mental illness.

### 3.2 Assessment scores

The measure summary scores for the participants are displayed in Table 1. As expected, paranoid thoughts decrease with age, *r* = − 0.28, p < 0.001. Experiential reasoning total scores did not vary by educational level, *F* (3, 495) = 0.41, *p* = 0.744, but rational reasoning total scores were lowest in those with the lowest education and highest in those with postgraduate degrees, *F* (3, 495) = 13.41, *p* < 0.001.

### 3.3 Associations with paranoid ideation

Table 2 displays the associations of the Paranoid Thoughts Scale with the measures. Higher levels of paranoid thoughts were associated with greater experiential reasoning and less analytic reasoning. The experiential and rational scales did not significantly correlate with each other. Paranoid thoughts were also associated with the presence of neuroticism. When experiential and rational scores were entered into a linear regression with paranoid thoughts as the dependent variable the model was significant, *F* (2,497) = 8.32, *p* < 0.001, Adjusted *R* Square = 0.03. Experiential reasoning, *B* = 0.02, standard error = 0.005, *p* = 0.004, and rational reasoning, *B* = − 0.02, standard error = 0.005, *p* = 0.003, each independently predicted paranoia. They remained significant predictors of paranoia when controlling for age, gender, ethnicity, and education. In an analysis of variance, there was no evidence in the prediction of paranoia scores of an interaction between experiential thinking and levels of neuroticism (*p* = 0.865).

**Fig. 1:**
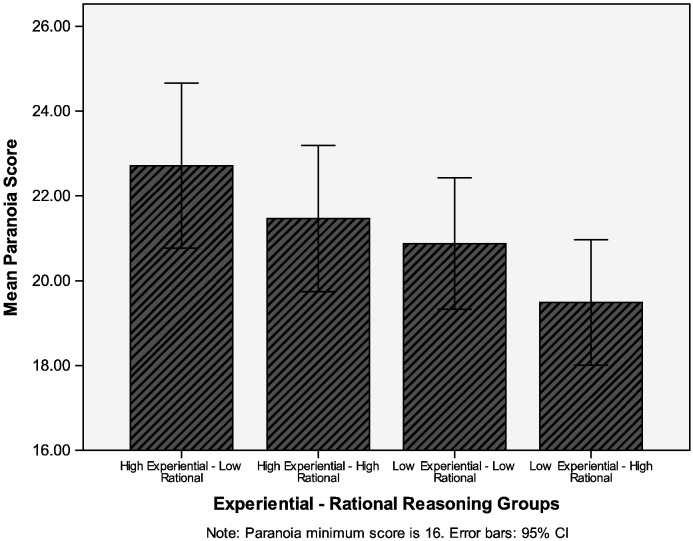
Mean paranoia scores by the interaction of experiential and rational reasoning styles.

Looking at the potential combinations of the two styles, the highest paranoia scores were in the individuals with high experiential/low rational scores, and the lowest paranoia scores were in those individuals with low experiential/high rational scores (see Fig. 1). The mean paranoia scores (SDs) for the groups were: high experiential/low rational (*n* = 126) = 22.7 (11.1); high experiential/high rational (*n* = 114) = 21.5 (9.3); low experiential/low rational (*n* = 131) = 20.9 (9.0); low experiential/high rational (*n* = 129) = 19.5 (8.5). There was an almost significant group difference in paranoia scores, *F* (3,496) = 2.53, *p* = 0.056, accounted for by the low experiential/high rational group scoring significantly lower than the high experiential/low rational group (*p* = 0.007). These groups also differed in levels of neuroticism, *F* (3, 496) = 7.66, *p* < 0.001. The low experiential/high rational group had lower levels of neuroticism than all three other groups (*p* < 0.05). In forming the four groups it is of interest that there was no association of scoring high or low in rational thinking and scoring high or low in experiential thinking, chi square (d.f. = 1) = 0.22, *p* = 0.636.

Interestingly we note that the individuals who reported a history of mental illness (*n* = 81) scored significantly higher for levels of paranoia and neuroticism but lower for levels of rational thinking and extraversion compared with the other study participants (*p* < 0.05). There was no difference in experiential thinking between those with a history of mental illness and the other participants (*p* = 0.905).

## 4 Discussion

In a large general population sample, the self-reported use of intuitive gut feelings was associated with higher levels of persecutory thinking, while the use of deliberative analytic thinking was protective. These are novel findings. The associations were small, explaining little of the variance in paranoia. However it should be borne in mind that it is rare for a self-report of general reasoning style to be linked to delusional ideation, and, moreover, the questionnaire items did not ask about reasoning in relation to the kinds of fearful feelings that are typically associated with delusions. A reliance on experiential thinking is likely to be significantly exacerbated in anxiety-provoking situations. Consistent with previous findings, rational and experiential thinking were found to be independent reasoning processes. The combination of experiential thinking without the check of rational thinking was most closely associated with paranoia.

There was a failure to find a predicted interaction between experiential reasoning and negative affective state. It was expected that a use of gut feelings in the context of anxiety would be more likely to result in paranoid interpretations of experience. However the assessment of neuroticism was very limited, comprising just two items, neither of which concerned the physiological components of anxiety. An expected main effect of neuroticism and paranoia was found. Higher levels of paranoia were associated with higher levels of neuroticism. This is consistent with the theoretical idea that paranoia and anxious affect are closely related via the process of threat anticipation (Freeman, 2007). Personality traits have not, to our knowledge, been specifically related before to persecutory thoughts. However it is quite plausible that reports of lowered mood state, less agreeableness, and lower extraversion are simply consequences of having paranoid thoughts. The association of paranoia with lower conscientiousness is less expected however. We resist speculation on this result until replication with a superior measure of personality.

The clear study weakness was the cross-sectional design. The associations could be explained by an unmeasured confounder, while the direction of the relationships between reasoning styles and paranoid thinking cannot be known. It would also have been of interest to include more clinical variables such as global functioning and emotional disorder. A further cross-sectional study in a group with delusions in the context of psychotic conditions such as schizophrenia is now warranted, although the optimal design would be to look longitudinally at whether the reasoning styles predict the persistence of delusional beliefs. Experimental or clinical studies testing whether manipulating therapeutically the type of reasoning style alters the course of delusions would be especially valuable (e.g. Freeman, 2011; Garety et al., 2011; Waller et al., 2011). It would also be informative to assess reasoning styles directly in relation to paranoid thoughts. Of interest here is that in two studies rational coping with suspicious thoughts has been linked with a lower frequency of paranoid thoughts (Freeman et al., 2005; Lincoln et al., 2010). There are also grounds to examine experiential and rational reasoning styles in relation to the delusion-relevant factors of jumping to conclusions and belief flexibility (So et al., 2012), working memory (Fletcher et al., 2011) and insomnia (Freeman et al., 2010a). It would also be intriguing to consider the neural correlates of the processing styles, for example the potential links of experiential reasoning with the insula (Singer et al., 2009). Clinically it is of note that cognitive therapy generally encourages analytic thinking in order to adjust problematic judgements and moderate the influence of subsequent experiential reasoning. It may prove helpful in cognitive approaches to psychosis to distinguish directly with patients between experiential and rational reasoning. Overall the study indicates that there may be merit in the application of the dual process framework to delusions.

## Figures and Tables

**Table 1:** The mean scores on the measures (*N* = 500).

Measure	Mean	S.D.	Minimum	Maximum
Paranoid Thoughts Scale Part B	21.1	9.5	16	76
Experiential Total	6.6	1.2	2.8	10.0
Rational Total	7.5	1.2	3.5	10.0
Experiential Engagement	3.3	0.7	1.1	5.0
Experiential Ability	3.3	0.7	1.3	5.0
Rational Engagement	3.8	0.7	1.7	5.0
Rational Ability	3.7	0.7	1.0	5.0
Extraversion	6.5	1.9	2.0	10.0
Neuroticism	6.1	2.1	2.0	10.0
Conscientiousness	7.3	2.1	2.0	10.0
Agreeableness	12.8	1.8	5.0	15.0
Openness	10.3	2.5	3.0	15.0

**Table 2:** Correlations between the measures (*N* = 500).

	Paranoid thoughts	Experiential Total	Rational Total
Experiential Total	0.12**		
	*p* = 0.006		
Rational Total	− 0.13**	0.04	
	*p* = 0.004	*p* = 0.411	
Experiential engagement	0.14**	0.91***	− 0.04
	*p* = 0.002	*p* < 0.001	*p* = 0.325
Experiential ability	0.09	0.91***	0.11*
	*p* = 0.055	*p* < 0.001	*p* = 0.012
Rational engagement	− 0.11*	0.04	0.89***
	*p* = 0.018	*p* = 0.424	*p* < 0.001
Rational ability	− 0.12**	0.03	0.90***
	*p* = 0.006	*p* = 0.496	*p* < 0.001
Extraversion	− 0.10*	0.19***	0.09*
	*p* = 0.025	*p* < 0.001	*p* = 0.049
Neuroticism	0.33***	0.08	− 0.23***
	*p* < 0.001	*p* = 0.085	*p* < 0.001
Conscientiousness	− 0.22***	− 0.03	0.17***
	*p* < 0.001	*p* = 0.447	*p* < 0.001
Agreeableness	− 0.25***	0.01	0.08
	*p* < 0.001	*p* = 0.844	*p* = 0.065
Openness	0.04	0.16***	0.33***
	*p* = 0.326	*p* < 0.001	*p* < 0.001

* *p* < 0.05, **p < 0.01 , ***p < 0.001.
